# Structural insights into drug development strategy targeting EGFR T790M/C797S

**DOI:** 10.18632/oncotarget.24113

**Published:** 2018-01-10

**Authors:** Su-Jie Zhu, Peng Zhao, Jiao Yang, Rui Ma, Xiao-E Yan, Sheng-Yong Yang, Jing-Wen Yang, Cai-Hong Yun

**Affiliations:** ^1^ Institute of Systems Biomedicine, School of Basic Medical Sciences, Peking University Health Science Center, Beijing 100191, China; ^2^ Department of Biophysics and School of Basic Medical Sciences, Peking University Health Science Center, Beijing 100191, China; ^3^ Beijing Key Laboratory of Tumor Systems Biology, School of Basic Medical Sciences, Peking University Health Science Center, Beijing 100191, China; ^4^ State Key Laboratory of Biotherapy, West China Hospital, West China Medical School, Sichuan University, Chengdu 610041, China; ^5^ Department of Prosthodontics, Peking University School and Hospital of Stomatology, Beijing 100081, China

**Keywords:** lung cancer, T790M/C797S, drug resistance, x-ray crystallography, structure-based drug design

## Abstract

Treatment of non-small-cell lung cancers (NSCLCs) harboring primary EGFR oncogenic mutations such as L858R and exon 19 deletion delE746_A750 (Del-19) using gefitinib/erlotinib ultimately fails due to the emergence of T790M mutation. Though WZ4002/CO-1686/AZD9291 are effective in overcoming EGFR T790M by targeting Cys797 via covalent bonding, their efficacy is again limited due to the emergence of C797S mutation. New agents effectively inhibiting EGFR T790M without covalent linkage through Cys 797 may solve this problem. We presented here crystal structures of EGFR activating/drug-resistant mutants in complex with a panel of reversible inhibitors along with mutagenesis and enzyme kinetic data. These data revealed a previously un-described hydrophobic clamp structure in the EGFR kinase which may be exploited to facilitate development of next generation drugs targeting EGFR T790M with or without concomitant C797S. Interestingly, mutations in the hydrophobic clamp that hinder drug binding often also weaken ATP binding and/or abolish kinase activity, thus do not readily result in resistance to the drugs.

## INTRODUCTION

The epidermal growth factor receptor (EGFR) is an important therapeutic target for non-small-cell lung cancers (NSCLCs) [1–5]. The 4-anilinoquinazoline based reversible EGFR tyrosine kinase inhibitors (TKIs) gefitinib and erlotinib have been demonstrated effective in treating advanced NSCLC with EGFR mutants [4–8]. Unfortunately, in most of the cases, NSCLCs with EGFR activating mutations eventually develop resistance to these drugs, and in at least half of the relapsed cases the resistance is caused by a single secondary mutation in EGFR that leads to Thr to Met substitution at residue 790 (T790M), the gate-keeper residue [9–12].

We previously showed that T790M mutation dramatically increases the ATP binding affinity of the oncogenic activating mutants and weakens the binding affinity of the drugs, hence reducing the potency of any ATP-competitive inhibitors [13]. This study suggested that to simply remove the steric hindrance of TKIs is not sufficient, and that a new drug must bind to T790M-bearing EGFR with much higher affinity than gefitinib/erlotinib binding to the activating mutants (EGFR L858R or Del-19) to overcome the enhanced ATP binding. Multiple strategies could be applied to fulfill this task, including covalent/irreversible inhibition (strategy I), super-high-potency non-covalent/reversible inhibition (strategy II) and non-ATP-competitive inhibition (strategy III, also known as allosteric inhibition). In cooperation with other groups, we and our colleagues have successfully developed an irreversible inhibitor WZ4002 (strategy I) [14] and an allosteric inhibitor EAI045 (strategy III) [15] to overcome drug-resistant EGFR mutations.

The irreversible inhibitors such as WZ4002 [14], AZD9291 [16] and CO-1686 [17] carry a Michael acceptor functional group to irreversibly alkylate a cysteine residue (C797) in the ATP binding site of EGFR, thus overcoming the problem of the enhanced ATP binding. In clinic studies, treatment with either CO-1686 or AZD9291 has resulted in more than 50% response rate in NSCLC patients bearing EGFR T790M, and are associated with substantially less skin toxicity than typically observed for first generation EGFR TKIs [17, 18]. Recently, AZD9291 was approved by FDA for the treatment of patients with metastatic EGFR T790M mutation-positive NSCLC who have progressed on or after EGFR TKI therapy [19].

Despite the success of AZD9291 in clinical trials, it remains a deep worry that patients receiving the treatment may ultimately develop acquired resistance to this (and other third-generation) new agents. Indeed, mutations L718Q, L844V and C797S were identified in drug resistant clones after N-ethyl-N-nitrosourea (ENU) mutagenesis screen in EGFR mutant (sensitizing alone or with concurrent EGFR T790M) Ba/F3 cells in a lab research. Importantly, EGFR C797S cause resistance to all three tested third-generation inhibitors WZ4002, CO-1686 and AZD9291; cells containing an EGFR sensitizing mutation, Del-19 or L858R, in conjunction with L718Q, L844V or C797S retain sensitivity to quinazoline based EGFR inhibitors gefitinib and afatinib [20]. More recently, the EGFR C797S mutation was identified in cell-free plasma DNA from subjects with advanced lung cancer whose tumors had developed resistance to AZD9291, proving that the C797S drug-resistance mutation is clinically relevant [21], and L718Q mutation was also identified in a 71-year old woman with advanced lung adenocarcinoma harboring the L858R mutation who became resistant to AZD9291 [22].

Most recently, we reported the discovery of EAI045, a novel inhibitor of EGFR that can overcomes EGFR L858R/T790M and L858R/T790M/C797S [15]. The EAI compounds were developed through high-throughput screening as allosteric inhibitors purposely to overcome EGFR T790M (strategy III), and later found effective to T790M/C797S, too. However, EAI045 must be used in combination with Cetuximab, and it is less effective to T790M/C797S with concurrent exon-19 deletion (Del-19) mutations. Hence EAI045 may not be an optimal agent to treat all EGFR C797S-related drug-resistant lung cancers, and new agents to inhibit EGFR L858R/T790M/C797S and Del-19/T790M/C797S, ideally without the help of a second agent such as Cetuximab, are needed.

Since C797S prevents covalent binding, the super-high-potency non-covalent/reversible inhibition strategy (strategy II) may apply in the development of new agents to overcome EGFR T790M/C797S. In a previous effort to discover reversible inhibitors targeting EGFR T790M mutation, a new compound, SKLB1206, was developed by Pan *et al.* [23]. SKLB1206 is a member of the SKLB compounds series based on the *N*^8^-phenyl-9*H*-purine-2,8-diamine scaffold targeting EGFR activating and T790M Mutants, ErbB2, ErbB4, and VEGFR2. Structure-activity relationships (SAR) studies on the SKLB compounds revealed that substitution at the N-9 position of the 9H-purine ring by hydrophobic groups could generally enhance the potency of the compound and that only substituent of suitable size and geometry was optimal for binding to EGFR [24].

In the presented work here, we studied the binding mode of the SKLB compounds to EGFR kinase by solving their complex crystal structures. These data suggested the existence of a “hydrophobic clamp” in the ATP-binding pocket of the EGFR kinase that might not be fully exploited in previous drug discovery studies. Interaction between the “hydrophobic clamp” and the compounds can well explain the SKLB SAR data. The hydrophobic clamp model can also explain the previously observed phenomenon that WZ4003 (a non-covalent analogue of WZ4002) binds to EGFR rather weakly, and explain why the newly discovered L718Q and L844V mutants are resistant to WZ4002 and CO-1686. More importantly, our studies indicated a new strategy to design high affinity reversible EGFR inhibitors without covalent binding through Cys 797, which might provide a new way to overcome both L858R/T790M and Del-19/T790M with or without concomitant C797S mutation. In the present work, we also provided mutagenesis and enzyme kinetic data to assess a panel of hydrophobic clamp mutations that might incur resistance to new agents relying on the hydrophobic clamp structure, and found that mutations weakening the binding of these agents in most cases also weaken the binding of ATP or may abolish the tumorigenic potency of the mutant EGFR, thus may not readily cause resistance to these agents.

## RESULTS

### *In vitro* structure-activity relationship (SAR) study on the SKLB purine core N-9 position

**Figure 1:**
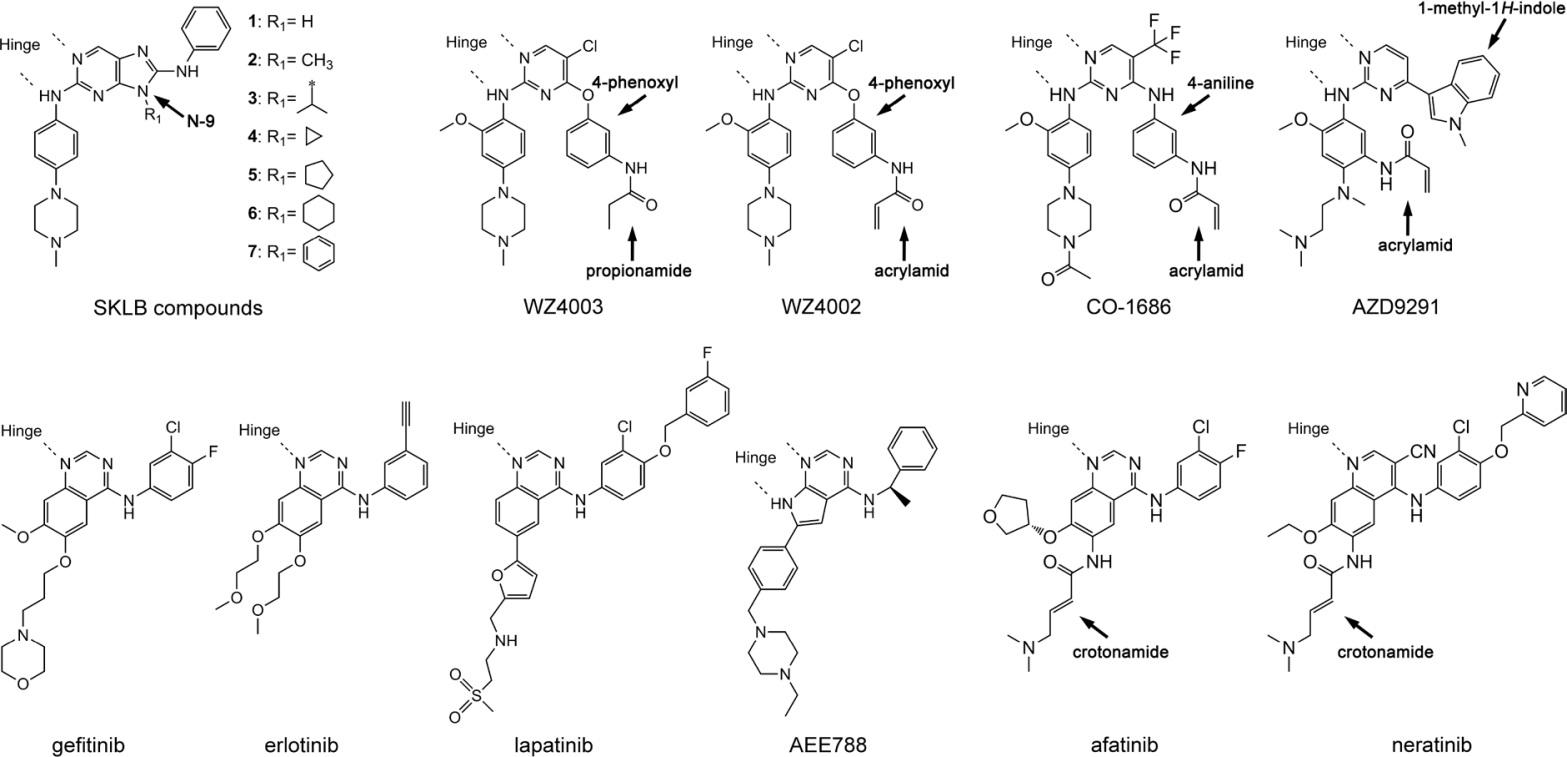
Chemical structures of EGFR TKIs discussed in this report

Previous SAR data on cell lines indicated that the size and shape of the hydrophobic substituent at the N-9 site of the SKLB compounds (Figure 1) play important roles to determine the potency of the compounds, and that cyclopentyl is an optimal substituent to achieve the highest potency [24]. Since drug efficacy in inhibiting cell proliferation depends on many issues including drug-binding affinity to the target kinase, cross-activity against other targets and cell membrane permeability of the compound *etc*., the aforementioned SAR data in the cell line studies do not necessarily mean that the drug binding potency to EGFR depends on size and shape of the N-9 substituent in the same way. Therefore, we determined the IC_50_ values of the SKLB compounds to inhibit purified EGFR L858R/T790M double mutant and L858R single mutant kinases *in vitro*. As is shown in Table 1, introduction of methyl group on 9-site of purine does not significantly change the inhibition potency relative to 1. Nevertheless, substitution of isopropyl (3) and cyclopropyl (4), both containing three carbons, considerably increases the inhibitory activity against EGFR L858R/T790M more than 10 times compared with that of 1 and 2. Increasing the size of the hydrophobic group to cyclopentyl (5) leads to a significant increase of the inhibitory activity against EGFR L858R/T790M to about 1000 times compared with that of 1. However, a further increase of the size of the substituent, such as cyclohexyl (6), and phenyl (7), leads to decreased bioactivity relative to cyclopentyl, implying that cyclopentyl presents the most suitable substituent for bioactivity. Interestingly, changing the hydrophobic substituent at the N-9 position also affects the inhibitory potency toward EGFR L858R single mutation and cyclopentyl remains the optimal substituent that inhibits L858R most potently; however the magnitude of the effect is much smaller relative to what is observed for the L858R/T790M double mutation. This observation indicates that to develop new agents against EGFR activating mutants with a concomitant L858R/T790M/C797S mutation conferring resistance to WZ4002, AZD9291 and CO-1686, the size and shape of the substituent at the N-9 position is critical design consideration.

**Table 1:** IC50 values of compounds 1-7 against human EGFR L858R/T790M and L858R mutants (IC50s are the average values from two independent experiments

ID	R_1_	EGFR (L858R/T790M) [nM]	EGFR (L858R) [nM]
1	H	522 ± 15	5 ± 3
2	CH_3_	458 ± 10	3 ± 2
3	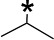	47 ± 2	5 ± 2
4	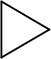	41 ± 3	2 ± 1
5	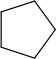	0.5 ± 0.4	0.4 ± 0.3
6	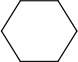	17 ± 2	4 ± 2
7	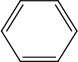	45 ± 5	1 ± 0.3

The asterisk (^*^) indicates the attachment point.

### Complex structures revealed a hydrophobic clamp structure in EGFR

**Figure 2:**
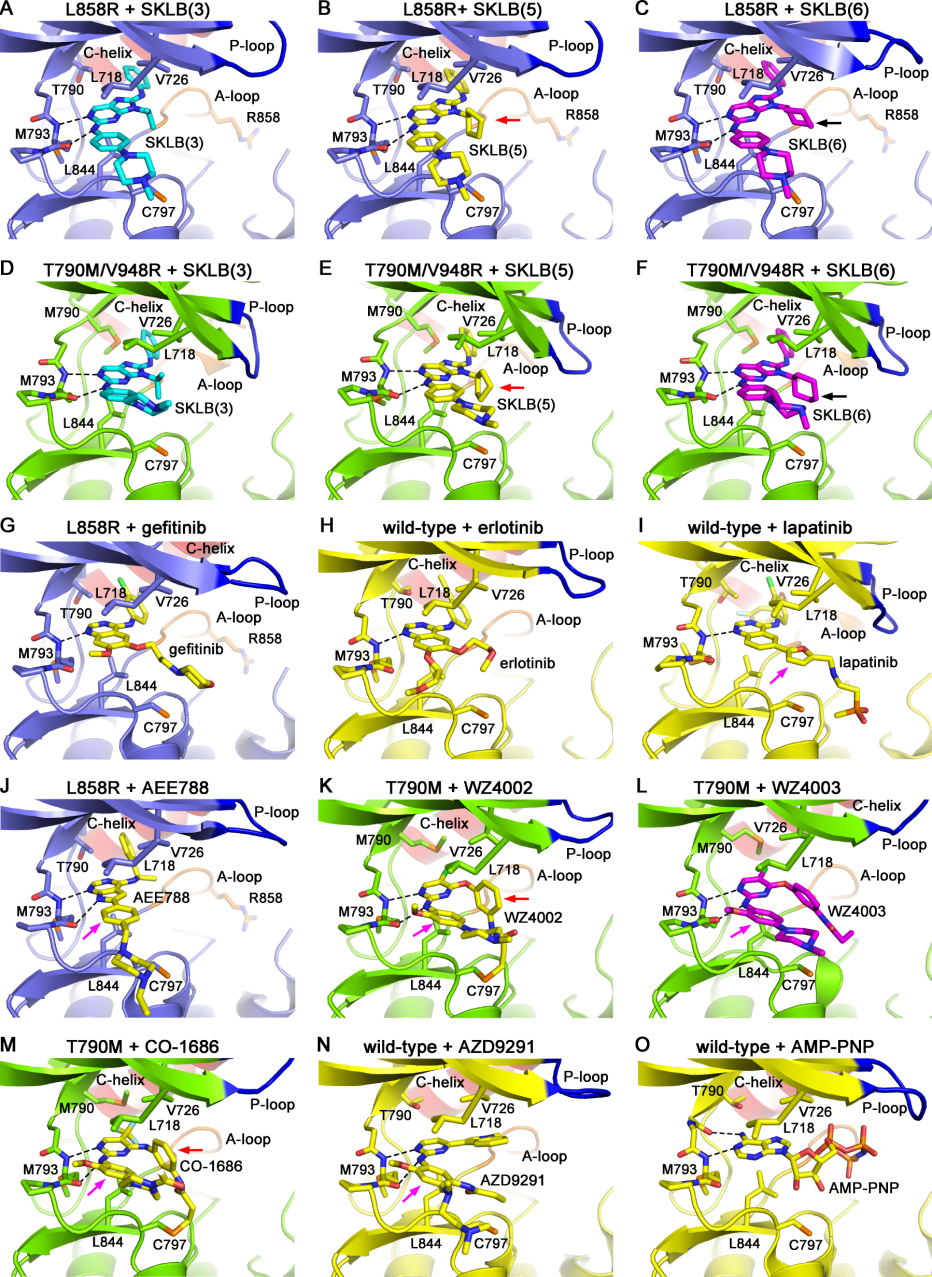
Crystal structures of EGFR mutants in complex with varied compounds (**A**) L858R + SKLB(3), (B) L858R + SKLB(5), (**C**) L858R + SKLB(6), (**D**) T790M/V948R + SKLB(3), (**E**) T790M/V948R + SKLB(5), (**F**) T790M/V948R + SKLB(6), (**G**) L858R + gefitinib (drawn from PDB 2itz), (**H**) wild-type + erlotinib (drawn from PDB 1m17), (**I**) wild-type + lapatinib (drawn from PDB 1xkk), (**J**) L858R + AEE788 (drawn from PDB 2itt), (**K**) T790M + WZ4002 (drawn from PDB 3ika), (**L**) T790M + WZ4003, (**M**) T790M + CO-1686 (drawn from PDB 5XDK), (**N**) wild-type + AZD9291 (drawn from PDB 4zau) and (**O**) wild-type + AMP-PNP (drawn from PDB 2itx, AMP-PNP is an analogue of ATP) complex structures are shown. The EGFR wild-type, L858R and T790M/V948R mutants are shown as yellow, slate and green cartoons, respectively. The compounds are shown as sticks. Hydrogen bonds are shown by black dashed lines. The black arrows in panels C and F indicate the lower part of the cyclohexyl (SKLB(6)) that does not fit to the interaction with Leu 844. The purple arrows in panels I, J, K, L, M and N indicate the moieties in lapatinib, AEE788, WZ4002, WZ4003, CO-1686 and AZD9291 that form weak hydrophobic interactions with the side-chain of Leu 718 but not Val 726. The red arrows in panels B, E, K and M indicate that the cyclopentyl in SKLB(5) and phenyl in WZ4002/CO-1686 are analogous to each other considering the interactions with the hydrophobic clamp.

In order to understand why the substituent at N-9 position dictates the inhibition potency of the SKLB compounds, we determined the crystal structure of EGFR kinase domain bearing L858R or T790M (in the form of T790M/V948R) in complex with SKLB(3), SKLB(5)and SKLB(6) (Figures 2A–2F, Supplementary Figures 1, 2, 3; Table 2, Supplementary Table 1, 2). Although the data resolutions of the L858R complex structures were modest, the electron densities of the compounds were clear (Supplementary Figures 1 and 3). The T790M/V948R+SKLB6 data completeness was about 89.4%, which might result in more noise peaks in the electron density maps. Fortunately, after inspecting the electron density maps (including the omit map), we found that the densities of the compound were clearly defined without ambiguity (Supplementary Figures 2C and 3F).

**Table 2:** A summary of the crystallographic data and refinement statistics^§^

mutation	compound	Structure ID	Resolution Range (Å)	R_sym_ (outmost)	Completeness, % (outmost)	R_work_/R_free_	RMSD bond length/angle
L858R	SKLB(3)	5X26	50.0–2.95	0.071 (0.413)	98.2 (97.9)	0.196/0.222	0.014/1.277
L858R	SKLB(5)	5X27	50.0–2.95	0.078 (0.416)	96.4 (98.7)	0.203/0.239	0.015/1.339
L858R	SKLB(6)	5X28	50.0–2.95	0.067 (0.426)	99.1 (98.1)	0.209/0.250	0.013/1.235
T790M/V948R	SKLB(3)	5X2A	50.0–1.85	0.074 (0.593)	98.0 (96.4)	0.174/0.219	0.023/1.890
T790M/V948R	SKLB(5)	5X2C	50.0–2.05	0.082 (0.482)	98.7 (98.0)	0.175/0.202	0.017/1.594
T790M/V948R	SKLB(6)	5X2F	50.0–2.20	0.106 (0.566)	89.4 (79.8)	0.202/0.240	0.014/1.325
T790M	WZ4003	5X2K	50.0–3.20	0.041 (0.415)	100.0 (100.0)	0.226/0.244	0.009/1.082

^§^ See Supplementary Table 1, 2 and 3 for additional statistics of data collection and structure refinement.

*R*_sym_*=*∑|*I*_i_ - <*I*_i_>|/∑*I*_i_, where *I*_i_ is the average intensity of symmetry-equivalent reflections.

*R*_work_=∑|F_o_-F_c_|/∑F_o_ where Fo and Fc are observed and calculated structure factor amplitudes, respectively.

*R*_free_ is the *R*_cryst_ for reflections excluded from the refinement.

Similar to the previously reported EGFR/inhibitor complexes [13, 14, 25–27], the SKLB compounds bind to EGFR in the ATP-binding cleft. The compounds closely associate with the hinge of the kinase, and form two hydrogen bonds with Met 793 amide and carbonyl. The 8-aniline ring occupies a hydrophobic pocket in the back of the ATP binding cleft, which is occupied by the 4-aniline ring in the 4-anilinoquinazolines (gefitinib and erlotinib, Figure 2G and 2H) and the phenylethanamine ring in AEE788 (an EGFR/ErbB2 and VEGFR dual family kinase inhibitor, Figure 2J) [28] when binding to EGFR. The isopropyl (SKLB(3)), cyclopentyl (SKLB (5)) and cyclohexyl (SKLB(6)) groups on the N-9 position of purine is sandwiched between Leu 718, Val 726 and Leu 844 side-chains, and are located within hydrophobic interaction distances to these side-chains (Figure 2A–2F). These residues engage the compounds from above and below through hydrophobic interactions like a clamp, thus we refer to them as a “hydrophobic clamp” structure of the kinase.

The strength of hydrophobic interactions depends on multiple issues including distance, area (size) and geometry (shape complementarity) of the hydrophobic groups/surfaces participating in the interaction. Too short or too long distances between the hydrophobic groups/surfaces result in steric hindrance or loss of hydrophobic interactions, respectively, both weakening the binding potency of the compounds; small or incompatible hydrophobic surface weaken the interactions, too. It is noted from the complex structure that the cyclopentyl in SKLB(5) fits best to the hydrophobic clamp due to its suitable size and conformation (Figure 2B, 2E). Compared to the cyclopentyl substituent on N-9 position of SKLB(5), the isopropyl in SKLB(3) is too small to provide sufficient hydrophobic surface for the interaction with the hydrophobic clamp (Figure 2A, 2D). Although cyclohexyl in SKLB(6) is large, its conformation does not fit optimally to the hydrophobic clamp. Cyclohexyl tends to adopt a chair-like conformation as is seen in the complex crystal structures presented here. Interestingly, the upper half of the cyclohexyl ring is located roughly in the same position as the upper parts of cyclopentyl and isopropyl in SKLB(5) and SKLB(3), respectively, likely because this position is well situated between Leu 718 and Val 726. However, for cyclohexyl, the chair-like conformation leaves the lower part of the ring positioned farther away from Leu 844 than cyclopentyl in SKLB(5) (indicated by black arrows in Figure 2C, 2F), thus resulting in weaker hydrophobic interactions with Leu 844. These subtle differences among SKLB 3, 5 and 6 in interaction with the hydrophobic clamp can therefore explain why SKLB(5) bearing cyclopentyl at the N-9 position binds to EGFR tighter than 3 and 6.

### Interaction between hydrophobic clamp and available ATP-competitive EGFR inhibitors

Previously reported complex crystal structures revealed the binding modes of varied EGFR tyrosine kinase inhibitors (TKIs) including gefitinib, erlotinib, lapatinib, AEE788 and WZ4002 [14, 25–27]. In view of the hydrophobic clamp structure that could facilitate the binding of ATP-competitive inhibitors, we inspected these complex structures and to our surprise, we found that most of these old-generation agents do not fully interact with the hydrophobic clamp from the structural pharmacology view.

The core scaffolds of the first-generation inhibitors (gefitinib, erlotinib, AEE788 and lapatinib *etc.*) are strip-shaped without a branched substituent at the position analogous to the N-9 position in the SKLB compounds. When binding to EGFR, these agents are placed roughly in parallel to the hinge of EGFR through hydrogen bonding (Figures 2G–2J). For gefitinib and erlotinib, their aromatic quinazoline core is placed within significant hydrophobic interaction distance only to the Leu 844 side-chain as is observed in the complex crystal structures, while Leu 718 and Val 726 do not contribute much to the hydrophobic interaction with the compounds since they are too far away from the quinazoline moiety. Lapatinib and AEE788 make more use of the hydrophobic clamp than do gefitinib and erlotinib. The furanyl ring in lapatinib and the phenyl ring in AEE788 (shown by purple arrows in Figure 2I and 2J) are located within hydrophobic interaction distance to the side-chain of Leu 718, but not to the side-chain of Val 726. Although inadequately exploiting the hydrophobic clamp, these first-generation agents still work well in treating NSCLCs harboring primary EGFR oncogenic mutations because these mutations (L858R and Del-19) markedly weaken the ATP binding affinity and thus open a therapeutic window for the inhibitors to bind to the kinase [27, 29]. However, since T790M significantly increase the binding affinity of the kinase to ATP, these agents would not robustly work on this mutation even if the steric hindrance between the Met 790 side-chain and the compounds are removed [13].

The newly developed non-covalent inhibitor SKLB(5), however, makes full use of the hydrophobic clamp due to the additional cyclopentyl rings attached to the core scaffold of the compounds (shown by red arrows in Figure 2B, 2E), which participates in direct interactions with Leu 844, Leu 718 and Val 726 side-chains. Interestingly, it was found in the structural analysis that the covalent inhibitor WZ4002, CO-1686 and AZD9291 also partly rely on the hydrophobic clamp to bind to EGFR, as 4-phenoxyl in WZ4002, 4-aniline in CO-1686 and 1-methyl-1*H*-indole in AZD9291 (Figure 1) directly interact with the hydrophobic residues (shown by red arrows in Figure 2K, 2M, 2N; also see Supplementary Figure 4) [14, 30, 31]. This observation can well explain the interesting finding that although L718Q and L844V led to resistance to WZ4002, they remained sensitive to quinazoline-based irreversible EGFR inhibitors afatinib and neratinib (which are similar to gefitinib or lapatinib, see Figure 1) [20].

### Interaction between hydrophobic clamp and WZ4002/WZ4003

Covalent inhibition has been shown to be an effective way to overcome the EGFR T790M drug-resistance mutation. In order to covalently link to the kinase through Cys 797, a branched chemical structure was used in WZ4002, in which a phenoxyl group was attached to the C-4 position of the pyrimidine core to carry the electrophilic acrylamide and bring it in close proximity to the side-chain of Cys 797 (Figure 1). Our previous structural studies revealed that the 4-phenoxyl moiety of WZ4002 passes through the hydrophobic clamp (Figures 2K) [14]. It is structurally analogous to the N-9 substituent of the SKLB compounds (Figure 1; Figure 2B, 2E and 2K; Supplementary Figure 4). Therefore, we hypothesized that the hydrophobic clamp structure should play some role in the binding of WZ4002 at least in the reversible binding stage as it does to the SKLB compounds; since a phenyl ring is not the optimal substituent for interacting with the hydrophobic clamp, the interactions between the hydrophobic clamp and WZ4002 (in the reversible binding stage) may not be the optimal.

**Figure 3:**
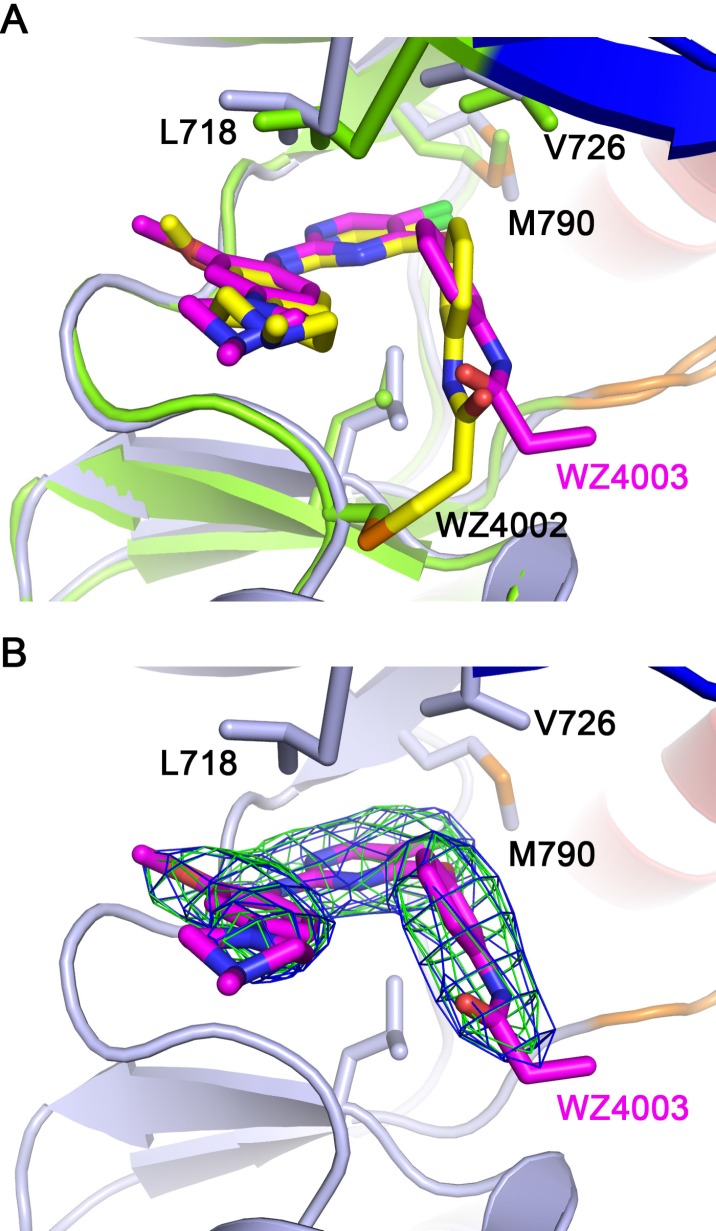
EGFR T790M + WZ4003 complex crystal structure (**A**) Superimposition of T790M + WZ4003 (reported here) and T790M + WZ4002 (drawn from PDB 3ika). The WZ4002 and WZ4003 bound EGFR structures are shown as green and light-blue cartoons, respectively. The key residues are shown as sticks with the same color scheme as the protein cartoons. WZ4002 and WZ4003 are shown as yellow and purple (carbon atoms) sticks, respectively. (**B**) Omit maps of WZ4003. The blue and green meshes indicate the 2Fo-Fc and Fo-Fc omit maps contoured at 1.0*σ* and 3.0*σ*, respectively. The omit maps were calculated after removing the coordinates of WZ4003 from the structure and refining the structure (simulated annealing beginning at 300K) to remove bias introduced by the WZ4003 coordinates.

In order to test the hypothesis that the hydrophobic clamp affects the binding of WZ4002 in the reversible binding stage, we determined the crystal structure of EGFR T790M in complex with WZ4003, a reversible analogue of WZ4002 (Figure 1; Figure 3). Interestingly and in agreement with our hypothesis, the 4-phenoxyl group of WZ4003 adopts a conformation obviously different from that observed in the T790M/WZ4002 complex [14]. In the EGFR T790M/WZ4003 complex, the phenoxyl group rotates about 30 degree compared to its counterpart in the EGFR T790M/WZ4002 structure, and brings the propionamide (analogous to the acrylamide in WZ4002) farther away from the side-chain of Cys 797 (Figure 3A). This observation indicated that the 4-phenoxyl group in WZ4002/WZ4003 is not optimal for bringing the propionamide/acrylamide in proximate to Cys 797 likely due to the interaction with the hydrophobic clamp, which may also explain the observation that WZ4003 binds to EGFR T790M rather weakly [14]. Although the 4-phenoxyl is not an optimal design for reversible binding of WZ4002 to EGFR, it still contributes to the binding rather than preventing it as was indicated by the observation that a phenyl at this position (such as SKLB(7)) enhances the binding of the compounds to EGFR comparing to no substation (SKLB(1)) or methyl (SKLB(2)) [24].

It is then deduced that mutations of the hydrophobic clamp may affect the binding affinity of WZ4002 to EGFR presumably in the reversible binding stage, and in some cases results in resistance to this agent, which would explain why L718Q and L844V mutations render EGFR T790M resistant to WZ4002 *in vitro* [20]. It is clear that Gln with a hydrophilic side-chain at residue 718 would not fit to the interaction with the WZ4002 phenoxyl. While in the case of L844V, although valine is a hydrophobic residue, it does not fit optimally to the interaction with the WZ4002 phenoxyl, because valine side-chain is much shorter than leucine side-chain, resulting in too long distance to the WZ4002 phenoxyl group for strong hydrophobic interaction.

### Impact of hydrophobic clamp mutations on SKLB(5) drug efficacy

Since hydrophobic interactions are sensitive to distance, surface area and geometry, we hypothesized that mutations of the hydrophobic clamp (L718X, V726X and L844X) may affect the binding affinity of any inhibitor that interacts with it, such as the SKLB compounds. However, efficacy of the ATP-competitive inhibitors is not solely determined by the binding affinity of the compounds, but also by ATP binding affinity. Therefore the ATP binding affinity should be considered, too. What’s more, certain mutations may interfere with the kinase function, e.g. shutting-down the activity of the kinase. These mutations may not result in drug-resistance in clinic even if they abolish the binding of the drugs. Therefore we sought to learn how some representative hydrophobic clamp mutations may interfere with drug binding, ATP binding and kinase activity of EGFR.

We tried to express a panel of EGFR hydrophobic clamp mutations on the background of L858R/T790M double mutation, i.e. L858R/T790M plus L718Q, L718N, L718V, L718F, V726T, V726A, V726F, V726L, L844N, L844V and L844F in sf9 insect cells for kinetic studies. Unfortunately, several of these triple mutants were not well-behaved and could not be purified to homogeneity, including L718Q and L844V, the two mutants that were found to be resistant to WZ4002 and CO-1686 in a lab mutagenesis study [20]. Finally we were able to prepare only five triple mutants, i.e. L858R/T790M/L718F, L858R/T790M/L718V, L858R/T790M/V726F, L858R/T790M/V726T and L858R/T790M/L844F. We then studied the kinetics of these mutants. L858R/T790M/C797S, a confirmed triple mutation conferring resistance to AZD9291/WZ4002/CO-1686 in which the third mutation C797S is not from the hydrophobic clamp of EGFR, was also included in our assays.

Consistent with our prediction, most of the hydrophobic clamp mutants remarkably weakened the binding affinity of SKLB(5), which partly relies on hydrophobic interactions with the hydrophobic clamp structure to bind to the kinase, while the non-hydrophobic-clamp mutant C797S did not alter the binding affinity of the compound much since it does not rely on covalent linkage with Cys 797 to bind to the kinase (Table 3). Interestingly, L718V mutation enhanced the binding of SKLB(5), indicating that the Valine side-chain provided even better hydrophobic interaction than the Leucine side-chain to interact with the cyclopentyl of SKLB(5).

**Table 3:** Inhibition constants (*K*_i_) and predicted drug efficacy (shown by *K*_i_/*K*_m_) of SKLB(*5*) to representative EGFR hydrophobic clamp mutants

Mutants	K_i_ (nM)	K_m_, _ATP_ (μM)	K_i_/K_m_ (×10-3)
L858R/T790M	3.2	11.0 ± 0.3	0.3
+L718F	63.3	22.3 ± 3.7	2.8
+L718V	1.4	43.7 ± 1.1	0.03
+V726F	84.9	113.4 ± 4.0	0.7
+V726T	34.4	17.2 ± 1.3	2.0
+L844F	80.1	121.1 ± 8.2	0.7
+C797S	7.0	13.2 ± 0.4	0.5

**Figure 4:**
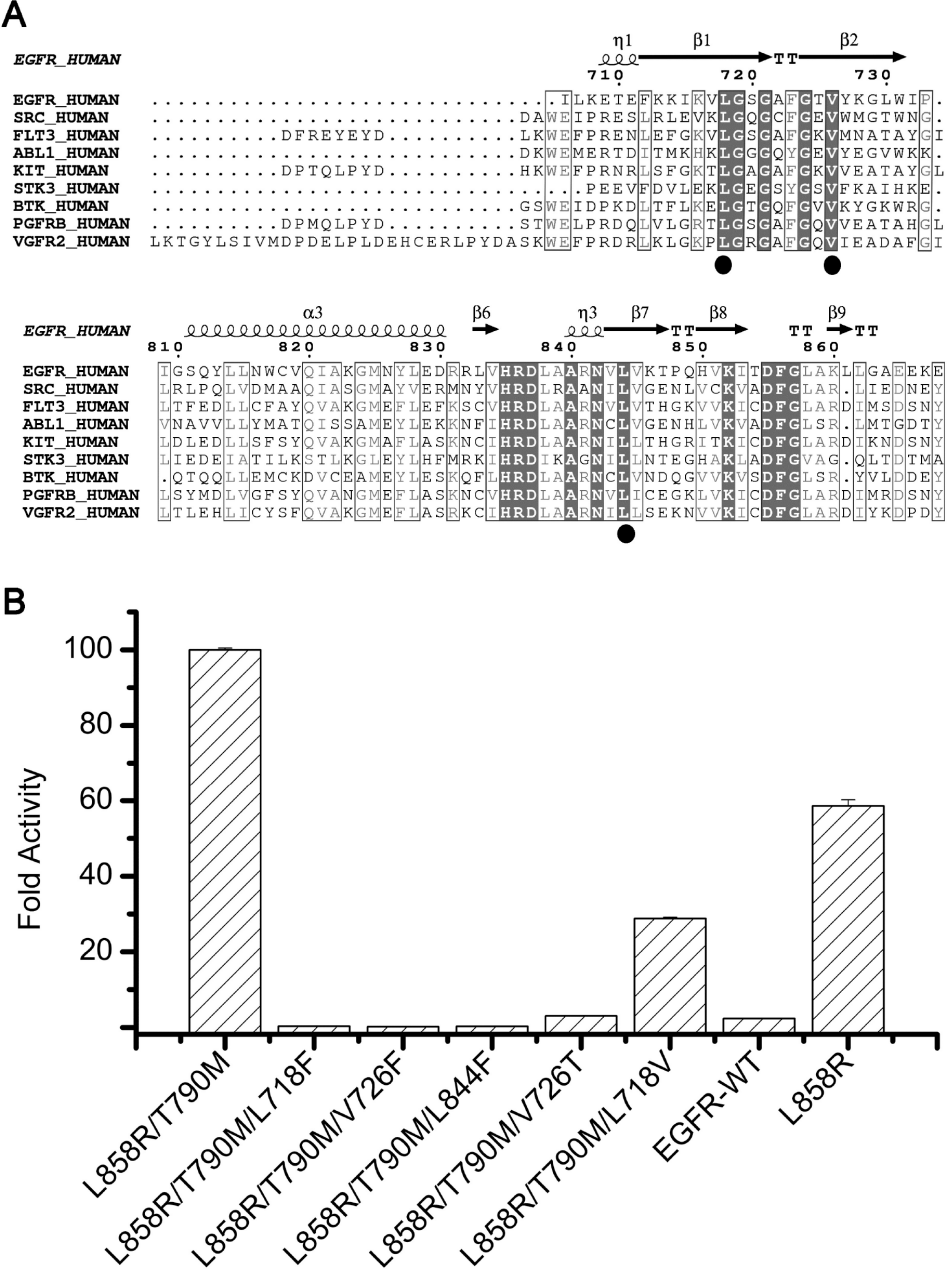
Impact of the hydrophobic clamp mutations on EGFR kinase activity (**A**) Sequence alignment of EGFR and other important kinase enzymes discussed in this report. Residues of the hydrophobic clamp are denoted by ovals. (**B**) Comparison of the activity of the wild-type, L858R/T790M and L858R/T790M plus the hydrophobic clamp mutants. The fold activity of wild-type and mutant enzymes was calculated by determining the *k*_cat_ for each protein with saturating ATP and 5mM ENAEYLRVA peptide substrate and dividing by the *k*_cat_ for the wild-type enzyme. Error bars indicate the standard deviation of the triplicate measurements. The active enzyme concentrations of every kinase samples were determined by titrating the protein samples with the tight-binding inhibitor SKLB(5) (see Methods).

For ATP-competitive inhibitors, the efficacy of the drugs must be evaluated by comparing the relative binding affinity of the drugs and ATP to EGFR, i.e. *K*_i_/*K*_m_, as was indicated in our previous studies (*K*_i_, the inhibition constant; *K*_m_, the Michaelis-Menton constant) [27]. Interestingly, most hydrophobic clamp mutations not only weakened the binding affinity of the compounds, but also weakened the binding affinity of ATP, among which the most remarkable ones were L858R/T790M/V726F and L858R/T790M/L844F (Table 3). This observation is not surprising, since the hydrophobic clamp structure is rather conserved in kinases (Figure 4A) and it interacts with ATP (Figure 2O), which indicates that any mutation in the hydrophobic clamp that weakens the drug binding may also affect ATP binding, as was observed in our studies. The overall effect of these mutations on drug binding and ATP binding was that no significant resistance to SKLB(5) was observed in our study.

### Impact of the hydrophobic clamp mutations on EGFR kinase activity

Inspired by the observation that the hydrophobic clamp mutations may weaken ATP binding, we sought to ask if these mutations may also impair kinase activity. The idea is that if a mutation turns off the kinase activity, even if it confers resistance to the drug, it would be of no harm in clinic. We therefore measured the activity (in terms of *k*_cat_) of the purified L858R/T790M/L718F, L858R/T790M/L718V, L858R/T790M/V726F, L858R/T790M/V726T, L858R/T790M/L844F, L858R and EGFR-WT (Figure 4B). Interestingly, according to the data, the introduction of most mutations led to a significant decrease of kinase activity. In particular, mutations L858R/T790M/L718F, L858R/T790M/V726F, L858R/T790M/L844F and L858R/T790M/V726T decreased the activity of the kinase to similar to or lower than that of the wild-type EGFR, thus potentially turned off the oncogenic potential of these mutations. Notably, although L718F and V726T could render EGFR L858R/T790M less sensitive to SKLB(5), they also significantly destroyed the activity of the kinase. Therefore it is deduced that these mutations, although may render cells resistant to the drugs *in vitro*, would not readily cause disease relapse in clinic.

## DISCUSSION

Treatment of NSCLCs driven by EGFR activating mutations including L858R and exon-19 deletions using anti-EGFR TKIs are effective in the beginning but ultimately fails due to the emergence of drug-resistance. Our previous studies on the major drug-resistant EGFR T790M mutation indicated that the mutation not only weakens drug binding through steric hindrance, but enhances ATP binding [13]. Therefore to simply modify the inhibitors to avoid the steric hindrance with Met 790 side-chain is not enough. A new drug to overcome T790M must gain substantial potency toward EGFR to compete off ATP binding. The third-generation drugs including AZD9291, CO-1686 and WZ4002, achieved this goal through covalent linkage to the chemically active Cys 797 side-chain, and would therefore lose their activities to EGFR if this residue is mutated. Under this situation, new agents not depending on covalent linkage through Cys 797 (reversible inhibition) are demanded. What’s more, the new agents still have to (1) specifically target the EGFR mutants harboring T790M and (2) very potently bind to the EGFR kinase to compete off ATP substrate. The studies on the third-generation drugs have accumulated a lot of knowledge about how to design the compounds to gain specificity targeting the T790M mutation, while how to significantly increase the drug binding potency to EGFR not depending on the covalent linkage remain a problem.

Inspired by the previous SAR studies on the SKLB compounds, we sought to answer why the N-9 substituents of these compounds dictate the inhibition/binding potency toward EGFR. Through analysis of a panel of EGFR+SKLB complex crystal structures we identified L718, V726 and L844 as the key residues to directly interact with the N-9 substituents of SKLBs, which explained why these substituents significantly affect the inhibition potency/binding affinity of SKLBs to EGFR. We named this structure as “hydrophobic clamp” because these hydrophobic residues engage the compounds from both sides like a “clamp”. L718 and V726 are located in the *β*-sheet adjacent to the P-loop in N-lobe, while L844 is located in C-lobe (Figure 2). Since previous structural studies have revealed that considerable P-loop conformational changes and N-lobe/C-lobe relative orientation changes mainly occur in EGFR kinase structures in active versus inactive states, we intentionally studied two major types of the EGFR kinase structures, i.e. the active conformation structures (the L858R structures, Figure 2A, 2B, 2C) and the inactive conformation structures (the T790M/V948R structures, Figure 2D, 2E, 2F). These structures clearly showed that no matter in active or inactive conformations, the hydrophobic clamp residues retain direct and close engagement with the compounds.

Exploiting the hydrophobic clamp structure described in this study would potentially serve as a new approach to improve the drug binding affinity of any ATP-competitive inhibitors. Through careful tailoring the inhibitor structure to fully exploit the hydrophobic clamp structure, it is possible to dramatically increase the drug binding potency to the kinase as was shown in the SAR studies on the SKLB compounds [24]. It is noted that the hydrophobic clamp structure described here is not only found in EGFR, but also found in many other popular kinase targets such as Src, Abl, ALK, KIT, FLT3, BTK and PDGFRb *etc.* (Figure 4A). This is not surprising since the hydrophobic clamp structure is involved in ATP binding, but developing new agents making use of this structure may cause problem in drug selectivity. The similar problem has been handled in the development of the third-generation drugs (WZ4002/CO-1686/AZD9291) targeting Cys 797 because several other kinases have a cysteine residue analogous to Cys797 in EGFR. It turned out that after careful designing the chemical structure of the inhibitors, the specificity problem could be solved. The knowledge gained in the development of the third-generation drugs would therefore potentially be applied in the design of new hydrophobic-clamp-dependent reversible inhibitors to improve their selectivity against the EGFR mutations.

In this study we also managed to evaluate, through mutagenesis and enzyme kinetic assays, if the hydrophobic clamp mutations would readily cause resistance to any drugs that depending on the hydrophobic clamp to bind to EGFR. Our data showed that most of such mutations studied in our work not only weakened the drug binding, but also weakened ATP binding, thus did not result in significant resistance to the drug; what’s more, most of these mutations also very significantly decrease the kinase activity, potentially turning off its oncogenic potency. On the other hand, though the hydrophobic clamp mutations, namely L718Q and L844V, have been shown to be resistant in cell line studies [20], they seem to be seldom seen in patients compared to C797S. We therefore speculate that although the hydrophobic clamp mutations might still be a new source of drug-resistance, the problem may not be so stringent as is expected. Taken together, the information gained from these studies may be helpful in directing the development of next generation drugs to overcome the EGFR L858R/T790M/C797S and Del-19/ T790M/C797S drug-resistance mutations.

## MATERIALS AND METHODS

### Purification of recombinant proteins

Constructs spanning residues 696-1022 of the human EGFR and bearing the mutations were expressed and purified using a baculovirus/insect cell system as described [27]. Baculovirus stocks expressing the different EGFR mutants were used to infect Sf9 insect cells. After harvesting, the insect cells were broken by sonication in lysis buffer (20 mM Tris, 150 mM NaCl, 3 mM KCl, 1% glycerol, 1 mM PMSF, 1 mM TCEP, 20 mM Imidazole, pH 8.0) supplemented with a protease inhibitors cocktail. Clarified cell lysates were incubated with chelating sepharose beads charged with Ni2+ (GE Healthcare) for 2 hours at 4°C and proteins eluted with elution buffer (20 mM Tris, 500 mM NaCl, 1% gycerol, 0.5 mM TCEP, 300 mM Imidazole, pH 8.0). And then the eluted protein were concentrated and applied to gel-filtration chromatography using a Superdex 200 column (GE Healthcare) to further purify the proteins. Purified proteins were dispensed into aliquots, flash-frozen in liquid nitrogen and stored at −80*°*C.

### Crystallization and structure determination

Crystals used in this study were prepared by hanging drop vapor diffusion. The reservoir solution for growing the inhibitor-free L858R crystals was 0.1M Hepes pH7.8, 40% PEG400, 0.15M NaCl, 5mM tris(2-carboxyethyl)-phosphine (TCEP). The SKLB compounds were introduced into the inhibitor-free L858R crystals by soaking the crystals for 4 hours at room temperature in the crystallization buffer supplemented with 1mM compound. The reservoir solution for growing T790M/V948R crystals was 0.1M Bis-Tris 5.0, 22.5% PEG3350, 5mM TCEP in the presence of 1mM AMP-PnP and 10mM MgCl_2_. T790M/V948R naturally crystallized in two different crystal forms (C2 or P2_1_), both diffracting well. The soaking was done by first washing the crystals in the mother liquor (0.1M Bis-Tris 5.0, 22.5% PEG3350, 5mM TCEP) twice and then soaking the crystals in the soaking buffer (mother liquor supplemented with 1mM SKLB compound) for 4 hour. During the soaking, the soaking buffer was changed three times by transferring the crystals into fresh soaking buffer in order to completely replace AMP-PnP with the compounds. The reservoir solution for growing the inhibitor-free T790M crystals was 0.1M Glycine pH8.0, 38% PEG300, 0.1M NaCl, 5mM tris(2-carboxyethyl)-phosphine (TCEP). The compound WZ4003 was introduced into the inhibitor-free T790M crystals by soaking the crystals for 2 hours at room temperature in the crystallization buffer supplemented with 1mM compound.

Diffraction data of the L858R+SKLB(3), L858R+SKLB(5) and L858R+SKLB(6) complex crystals were collected on beamline ID24E at 100K at Argonne National Laboratory. Diffraction data of the T790M/V948R+ SKLB(3), T790M/V948R+ SKLB(5) and T790M/V948R+ SKLB(6) complex crystals were collected on the in-house x-ray diffraction system composed of a Bruker MicroStar X-ray generator and a Mar345dtb detector. Diffraction data of the T790M+WZ4003 complex crystals were collected on beamline BL19U1 at 100K at Shanghai Synchrotron Radiation Facility (SSRF). The diffraction data were processed using HKL3000 [32]. The L858R+SKLB, T790M/V948R+SKLB and T790M+WZ4003 complex structures were solved by molecular replacement with Phaser [33] using the previously determined EGFR L858R+ANP structure (PDB ID 2itv) [27], EGFR V948R structure (PDB ID 2GS7) [34] and T790M structure (PDB ID 2JIT) [13] as the search models, respectively. CNS [35] was then used to obtain a less biased model (by simulated-annealing) and calculate the sigmaA weighted 2Fo-Fc and Fo-Fc maps for manual inspection and adjustment. Repeated rounds of manual refitting and crystallographic refinement were then performed using COOT [36] and Phenix [37]. The inhibitor was modeled into the closely fitting positive Fo-Fc electron density and included in following refinement cycles. Topology and parameter files for the inhibitor were generated using PRODRG [38]. The crystal diffraction data and refinement statistics were summarized in Table 1 and Supplementary Table S1, S2 and S3. The L858R+SKLB(3), L858R+SKLB(5), L858R+SKLB(6), T790M/V948R+SKLB(3), T790M/V948R+ SKLB(5), T790M/V948R+ SKLB(6) and T790M+WZ4003 complex crystal structures have been deposited in Protein Data Bank (PDB) with entry IDs 5X26, 5X27, 5X28, 5X2A, 5X2C, 5X2F and 5X2K, respectively.

### Kinase inhibition assay

Kinase inhibition potency in Table 1 was measured using radiometric assay provided by Kinase Profiler service. Briefly, each kinase (5-10 mU) was incubated with a serially diluted solutions of compounds 1-7 in 25 μL reaction solutions. The reaction was initiated by adding the Mg-ATP mix. After incubation for 40 minutes at room temperature, the reaction was stopped by adding 5 μL of 3% phosphoric acid solution. 10 μL of the reaction was then spotted onto a Filtermat A and washed three times for 5 minutes in 75 mM phosphoric acid and once in methanol prior to drying and scintillation counting. ATP concentrations used in the assays were equal to the Km’s of the corresponding enzymes. Staurosporine was used as the positive control.

EGFR kinetic parameters in Table 3 were determined in triplicate using the ATP/NADH coupled enzyme assay method in a 96-well format as described [13]. The reaction mixture contained 1.0 mg/ml BSA, 2 mM MnCl_2_, 1 mM phospho(enol) pyruvic acid (PEP; Sigma-Aldrich; catalogue no. P7002), 2 mM TCEP, 0.1 M MOPS 7.4, 5 mM synthesized peptide ENAEYLRVA, 1/50 of the final reaction mixture volume of pyruvate kinase/lactic dehydrogenase enzymes from rabbit muscle (Sigma-Aldrich; catalogue no. P-0294), 0.5mM NADH. To determine *K*_m_, ATP at varied concentration was added last to start the reaction. Steady-state initial velocity data were drawn from the slopes of the A340 curves and fit to the Michaelis-Menten equation to determine V_m_ and *K*_m_. To assure that our derived *k*_cat_ parameters reflected concentrations of active enzyme, we determined the active enzyme concentration of every kinase preparation by titration of the samples with the tight binding inhibitor SKLB(5) (see below for the Morrison Equation).

The raw data to calculate *K*_i_ were dose response curves done by fixing ATP concentration and changing inhibitor concentration in the assays. The *K*_i_^app^ value was first determined using the Morrison Equation (for tight-binding substrate competitive inhibition):

$$\frac{v}{v_{0}}=1-\frac{\left(\left[E_{t}\right]+\left[I_{t}\right]+K_{i}^{app}\right)-\sqrt{{\left(\left[E_{t}\right]+\left[I_{t}\right]+K_{i}^{app}\right)}^{2}-4\left[E_{t}\right]\left[I_{t}\right]}}{2\left[E_{t}\right]}$$

and *K*_i_ was then calculated using

$$K_{i}^{app}=K_{i}\left(1+\frac{\left[ATP\right]}{K_{m}}\right)$$

All *K*_m_ and *K*_i_ values were determined using this method in triplicate, and the mean values and standard deviation were then calculated.

## SUPPLEMENTARY MATERIALS FIGURES AND TABLES



## References

[1] Paez JG, Janne PA, Lee JC, Tracy S, Greulich H, Gabriel S, Herman P, Kaye FJ, Lindeman N, Boggon TJ, Naoki K, Sasaki H, Fujii Y (2004). EGFR mutations in lung cancer: correlation with clinical response to gefitinib therapy. Science.

[2] Lynch TJ, Bell DW, Sordella R, Gurubhagavatula S, Okimoto RA, Brannigan BW, Harris PL, Haserlat SM, Supko JG, Haluska FG, Louis DN, Christiani DC, Settleman J (2004). Activating mutations in the epidermal growth factor receptor underlying responsiveness of non-small-cell lung cancer to gefitinib. N Engl J Med.

[3] Pao W, Miller V, Zakowski M, Doherty J, Politi K, Sarkaria I, Singh B, Heelan R, Rusch V, Fulton L, Mardis E, Kupfer D, Wilson R (2004). EGF receptor gene mutations are common in lung cancers from “never smokers” and are associated with sensitivity of tumors to gefitinib and erlotinib. Proc Natl Acad Sci U S A.

[4] Mok TS, Wu YL, Thongprasert S, Yang CH, Chu DT, Saijo N, Sunpaweravong P, Han B, Margono B, Ichinose Y, Nishiwaki Y, Ohe Y, Yang JJ (2009). Gefitinib or carboplatin-paclitaxel in pulmonary adenocarcinoma. N Engl J Med.

[5] Rosell R, Carcereny E, Gervais R, Vergnenegre A, Massuti B, Felip E, Palmero R, Garcia-Gomez R, Pallares C, Sanchez JM, Porta R, Cobo M, Garrido P (2012). Erlotinib versus standard chemotherapy as first-line treatment for European patients with advanced EGFR mutation-positive non-small-cell lung cancer (EURTAC): a multicentre, open-label, randomised phase 3 trial. The lancet oncology.

[6] Maemondo M, Inoue A, Kobayashi K, Sugawara S, Oizumi S, Isobe H, Gemma A, Harada M, Yoshizawa H, Kinoshita I, Fujita Y, Okinaga S, Hirano H (2010). Gefitinib or chemotherapy for non-small-cell lung cancer with mutated EGFR. N Engl J Med.

[7] Zhou C, Wu YL, Chen G, Feng J, Liu XQ, Wang C, Zhang S, Wang J, Zhou S, Ren S, Lu S, Zhang L, Hu C (2011). Erlotinib versus chemotherapy as first-line treatment for patients with advanced EGFR mutation-positive non-small-cell lung cancer (OPTIMAL, CTONG-0802): a multicentre, open-label, randomised, phase 3 study. The lancet oncology.

[8] Mitsudomi T, Morita S, Yatabe Y, Negoro S, Okamoto I, Tsurutani J, Seto T, Satouchi M, Tada H, Hirashima T, Asami K, Katakami N, Takada M (2010). Gefitinib versus cisplatin plus docetaxel in patients with non-small-cell lung cancer harbouring mutations of the epidermal growth factor receptor (WJTOG3405): an open label, randomised phase 3 trial. The lancet oncology.

[9] Pao W, Miller VA, Politi KA, Riely GJ, Somwar R, Zakowski MF, Kris MG, Varmus H (2005). Acquired resistance of lung adenocarcinomas to gefitinib or erlotinib is associated with a second mutation in the EGFR kinase domain. PLoS Med.

[10] Kwak EL, Sordella R, Bell DW, Godin-Heymann N, Okimoto RA, Brannigan BW, Harris PL, Driscoll DR, Fidias P, Lynch TJ, Rabindran SK, McGinnis JP, Wissner A (2005). Irreversible inhibitors of the EGF receptor may circumvent acquired resistance to gefitinib. Proc Natl Acad Sci U S A.

[11] Kobayashi S, Boggon TJ, Dayaram T, Janne PA, Kocher O, Meyerson M, Johnson BE, Eck MJ, Tenen DG, Halmos B (2005). EGFR mutation and resistance of non-small-cell lung cancer to gefitinib. N Engl J Med.

[12] Yu HA, Arcila ME, Rekhtman N, Sima CS, Zakowski MF, Pao W, Kris MG, Miller VA, Ladanyi M, Riely GJ (2013). Analysis of tumor specimens at the time of acquired resistance to EGFR-TKI therapy in 155 patients with EGFR-mutant lung cancers. Clin Cancer Res.

[13] Yun CH, Mengwasser KE, Toms AV, Woo MS, Greulich H, Wong KK, Meyerson M, Eck MJ (2008). The T790M mutation in EGFR kinase causes drug resistance by increasing the affinity for ATP. Proc Natl Acad Sci U S A.

[14] Zhou W, Ercan D, Chen L, Yun CH, Li D, Capelletti M, Cortot AB, Chirieac L, Iacob RE, Padera R, Engen JR, Wong KK, Eck MJ (2009). Novel mutant-selective EGFR kinase inhibitors against EGFR T790M. Nature.

[15] Jia Y, Yun CH, Park E, Ercan D, Manuia M, Juarez J, Xu C, Rhee K, Chen T, Zhang H, Palakurthi S, Jang J, Lelais G (2016). Overcoming EGFR(T790M) and EGFR(C797S) resistance with mutant-selective allosteric inhibitors. Nature.

[16] Cross DA, Ashton SE, Ghiorghiu S, Eberlein C, Nebhan CA, Spitzler PJ, Orme JP, Finlay MR, Ward RA, Mellor MJ, Hughes G, Rahi A, Jacobs VN (2014). AZD9291, an irreversible EGFR TKI, overcomes T790M-mediated resistance to EGFR inhibitors in lung cancer. Cancer discovery.

[17] Sequist LV, Soria JC, Gadgeel SM, Wakelee HA, Camidge DR, Varga A, Solomon BJ, Papadimitrakopoulou V, Jaw-Tsai SS, Caunt L, Kaur P, Rolfe L, Allen AR First-in-human evaluation of CO-1686, an irreversible, highly selective tyrosine kinase inhibitor of mutations of EGFR (activating and T790M). 2014 ASCO Annual Meeting.

[18] Janne PA, Ramalingam SS, Yang JCH, Ahn MJ, Kim DW, Kim SW, Planchard D, Ohe Y, Felip E, Watkins C, Cantarini M, Ghiorghiu S, Ranson M Clinical activity of the mutant-selective EGFR inhibitor AZD9291 in patients (pts) with EGFR inhibitor–resistant non-small cell lung cancer (NSCLC). 2014 ASCO Annual Meeting.

[19] Greig SL (2016). Osimertinib: First Global Approval. Drugs.

[20] Ercan D, Choi HG, Yun CH, Capelletti M, Xie T, Eck MJ, Gray NS, Janne PA (2015). EGFR mutations and resistance to Irreversible pyrimidine based EGFR inhibitors. Clin Cancer Res.

[21] Thress KS, Paweletz CP, Felip E, Cho BC, Stetson D, Dougherty B, Lai Z, Markovets A, Vivancos A, Kuang Y, Ercan D, Matthews SE, Cantarini M (2015). Acquired EGFR C797S mutation mediates resistance to AZD9291 in non-small cell lung cancer harboring EGFR T790M. Nature medicine.

[22] Bersanelli M, Minari R, Bordi P, Gnetti L, Bozzetti C, Squadrilli A, Lagrasta CA, Bottarelli L, Osipova G, Capelletto E, Mor M, Tiseo M (2016). L718Q Mutation as New Mechanism of Acquired Resistance to AZD9291 in EGFR-Mutated NSCLC. Journal of thoracic oncology.

[23] Pan Y, Xu Y, Feng S, Luo S, Zheng R, Yang J, Wang L, Zhong L, Yang HY, Wang BL, Yu Y, Liu J, Cao Z (2012). SKLB1206, a novel orally available multikinase inhibitor targeting EGFR activating and T790M mutants, ErbB2, ErbB4, and VEGFR2, displays potent antitumor activity both *in vitro* and *in vivo*. Molecular cancer therapeutics.

[24] Yang J, Wang LJ, Liu JJ, Zhong L, Zheng RL, Xu Y, Ji P, Zhang CH, Wang WJ, Lin XD, Li LL, Wei YQ, Yang SY (2012). Structural optimization and structure-activity relationships of N2-(4-(4-Methylpiperazin-1-yl)phenyl)-N8-phenyl-9H-purine-2,8-diamine derivatives, a new class of reversible kinase inhibitors targeting both EGFR-activating and resistance mutations. J Med Chem.

[25] Stamos J, Sliwkowski MX, Eigenbrot C (2002). Structure of the epidermal growth factor receptor kinase domain alone and in complex with a 4-anilinoquinazoline inhibitor. The Journal of biological chemistry.

[26] Wood ER, Truesdale AT, McDonald OB, Yuan D, Hassell A, Dickerson SH, Ellis B, Pennisi C, Horne E, Lackey K, Alligood KJ, Rusnak DW, Gilmer TM, Shewchuk L (2004). A unique structure for epidermal growth factor receptor bound to GW572016 (Lapatinib): relationships among protein conformation, inhibitor off-rate, and receptor activity in tumor cells. Cancer Res.

[27] Yun CH, Boggon TJ, Li Y, Woo MS, Greulich H, Meyerson M, Eck MJ (2007). Structures of lung cancer-derived EGFR mutants and inhibitor complexes: mechanism of activation and insights into differential inhibitor sensitivity. Cancer Cell.

[28] Traxler P, Allegrini PR, Brandt R, Brueggen J, Cozens R, Fabbro D, Grosios K, Lane HA, McSheehy P, Mestan J, Meyer T, Tang C, Wartmann M (2004). AEE788: a dual family epidermal growth factor receptor/ErbB2 and vascular endothelial growth factor receptor tyrosine kinase inhibitor with antitumor and antiangiogenic activity. Cancer Res.

[29] Carey KD, Garton AJ, Romero MS, Kahler J, Thomson S, Ross S, Park F, Haley JD, Gibson N, Sliwkowski MX (2006). Kinetic analysis of epidermal growth factor receptor somatic mutant proteins shows increased sensitivity to the epidermal growth factor receptor tyrosine kinase inhibitor, erlotinib. Cancer Res.

[30] Yan XE, Zhu SJ, Liang L, Zhao P, Choi HG, Yun CH (2017). Structural basis of mutant-selectivity and drug-resistance related to CO-1686. Oncotarget.

[31] Yosaatmadja Y, Silva S, Dickson JM, Patterson AV, Smaill JB, Flanagan JU, McKeage MJ, Squire CJ (2015). Binding mode of the breakthrough inhibitor AZD9291 to epidermal growth factor receptor revealed. Journal of structural biology.

[32] Minor W, Cymborowski M, Otwinowski Z, Chruszcz M (2006). HKL-3000: the integration of data reduction and structure solution--from diffraction images to an initial model in minutes. Acta Crystallogr D Biol Crystallogr.

[33] McCoy AJ, Grosse-Kunstleve RW, Adams PD, Winn MD, Storoni LC, Read RJ (2007). Phaser crystallographic software. Journal of applied crystallography.

[34] Zhang X, Gureasko J, Shen K, Cole PA, Kuriyan J (2006). An allosteric mechanism for activation of the kinase domain of epidermal growth factor receptor. Cell.

[35] Brunger AT, Adams PD, Clore GM, DeLano WL, Gros P, Grosse-Kunstleve RW, Jiang JS, Kuszewski J, Nilges M, Pannu NS, Read RJ, Rice LM, Simonson T, Warren GL (1998). Crystallography & NMR system: A new software suite for macromolecular structure determination. Acta Crystallogr D Biol Crystallogr.

[36] Emsley P, Cowtan K (2004). Coot: model-building tools for molecular graphics. Acta Crystallogr D Biol Crystallogr.

[37] Adams PD, Afonine PV, Bunkoczi G, Chen VB, Davis IW, Echols N, Headd JJ, Hung LW, Kapral GJ, Grosse-Kunstleve RW, McCoy AJ, Moriarty NW, Oeffner R (2010). PHENIX: a comprehensive Python-based system for macromolecular structure solution. Acta Crystallogr D Biol Crystallogr.

[38] Schuttelkopf AW, van Aalten DM (2004). PRODRG: a tool for high-throughput crystallography of protein-ligand complexes. Acta Crystallogr D Biol Crystallogr.

