# Supplementary material for: Structural insights into drug development strategy targeting EGFR T790M/C797S
Source: Oncotarget. 2018 Jan 10;9(17):13652–65. doi: 10.18632/oncotarget.24113 (PMC5862605; doi:10.18632/oncotarget.24113)
Supplement: Supplementary file 1 [file oncotarget-09-13652-s001.pdf]

Structural insights into drug development strategy targeting EGFR T790M/C797S

SUPPLEMENTARY MATERIALS

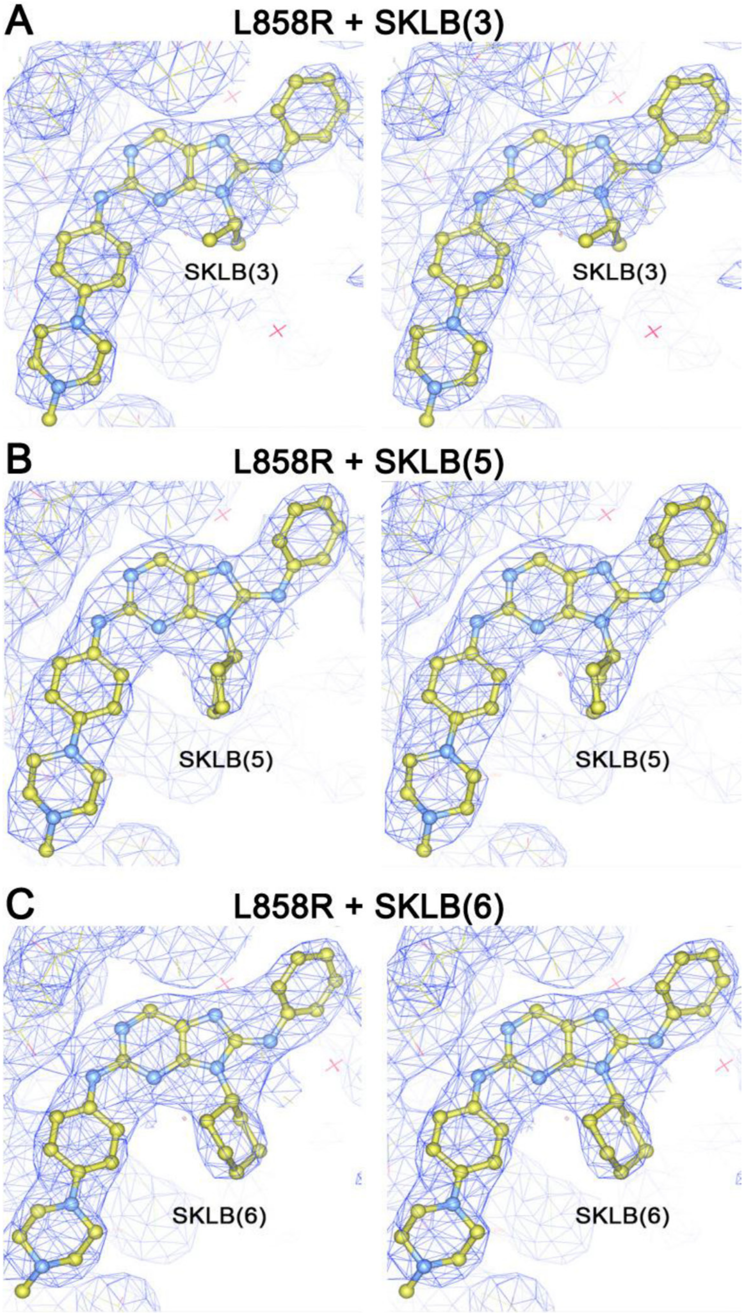

Supplementary Figure 1: Stereo views of the 2Fo-Fc electron density maps of SKLB compounds (contoured at 1.0σ) in the EGFR L858R + SKLB complex crystal structures. (**A**) L858R + SKLB(3). (**B**) L858R + SKLB(5). (**C**) L858R + SKLB(6).

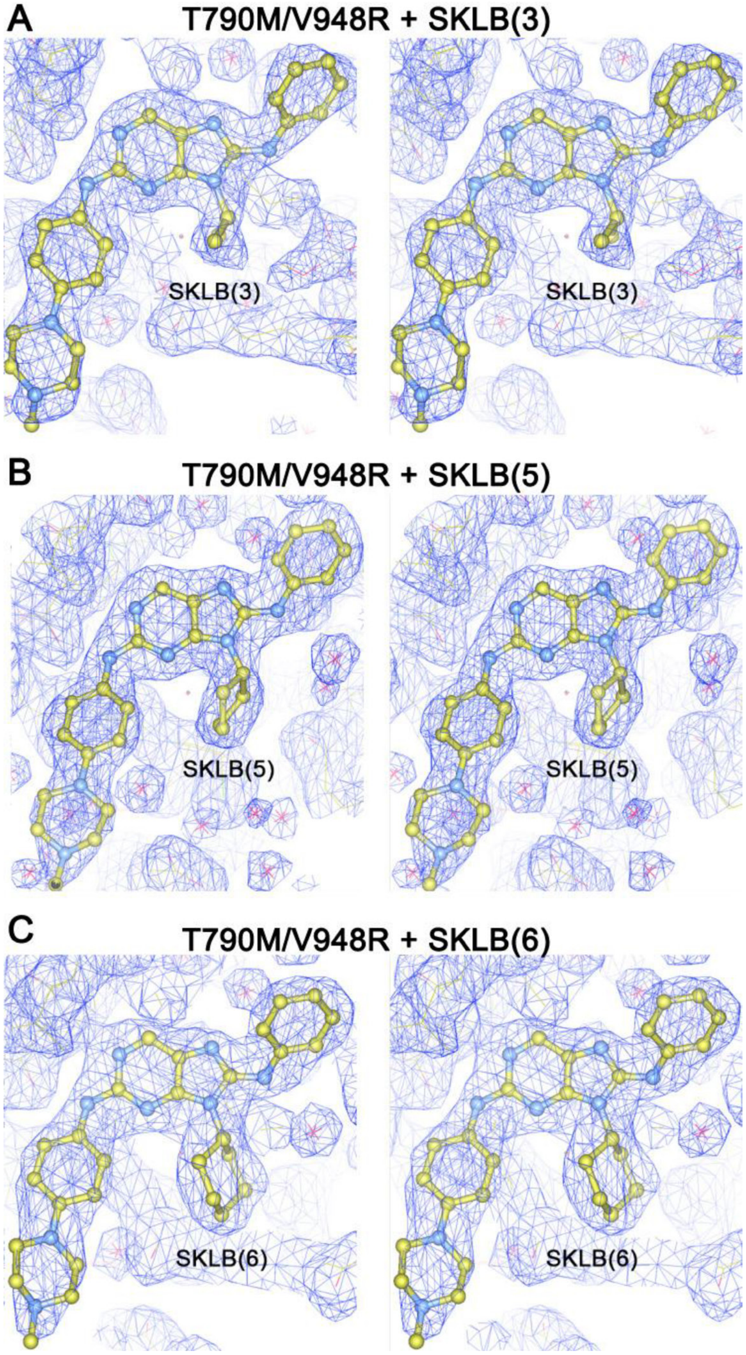

Supplementary Figure 2: Stereo views of the 2Fo-Fc electron density maps of SKLB compounds (contoured at 1.0σ) in the EGFR T790M/V948R + SKLB complex crystal structures. (**A**) T790M/V948R + SKLB(3). (**B**) T790M/V948R + SKLB(5). (**C**) T790M/V948R + SKLB(6).

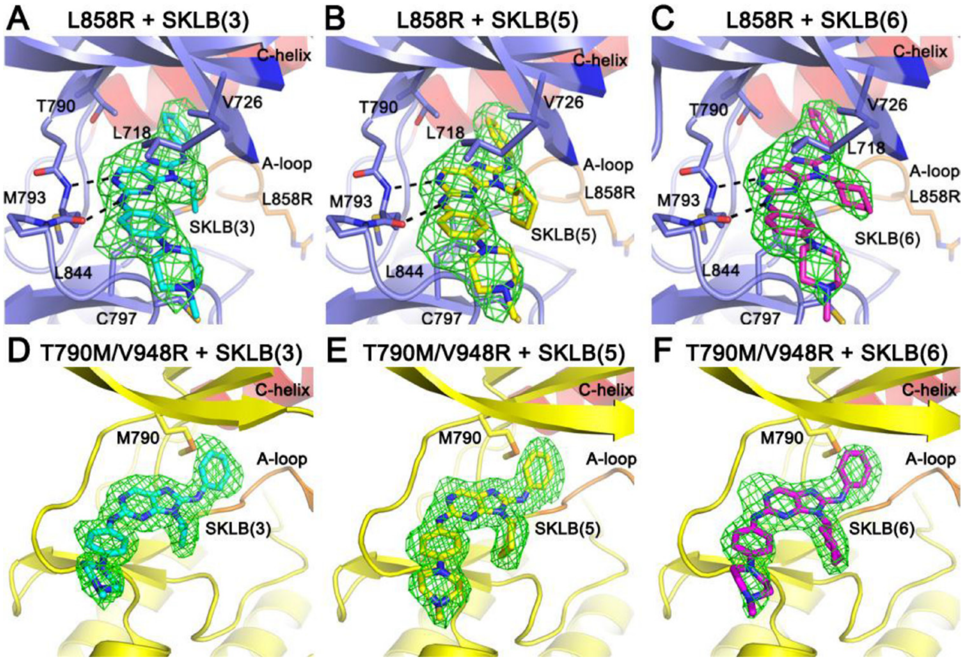

Supplementary Figure 3: The unbiased Fo-Fc maps (contoured at 2.0σ) for the EGFR+SKLB complex crystal structures. To calculate the unbiased omit maps, the coordinates of the SKLB compounds was removed from the refined structures and then the structures were refined by simulated-annealing using CNS to remove model bias before calculating the Fo-Fc electron density maps. (**A**) L858R + SKLB(3), (**B**) L858R + SKLB(5), (**C**) L858R + SKLB(6), (**D**) T790M/V948R + SKLB(3), (**E**) T790M/V948R + SKLB(5), (**F**) T790M/V948R + SKLB(6). The EGFR L858R and T790M/V948R protein structures are shown as slate and yellow cartoons, respectively. The compounds are shown as sticks. The Fo-Fc maps are shown as green meshes.

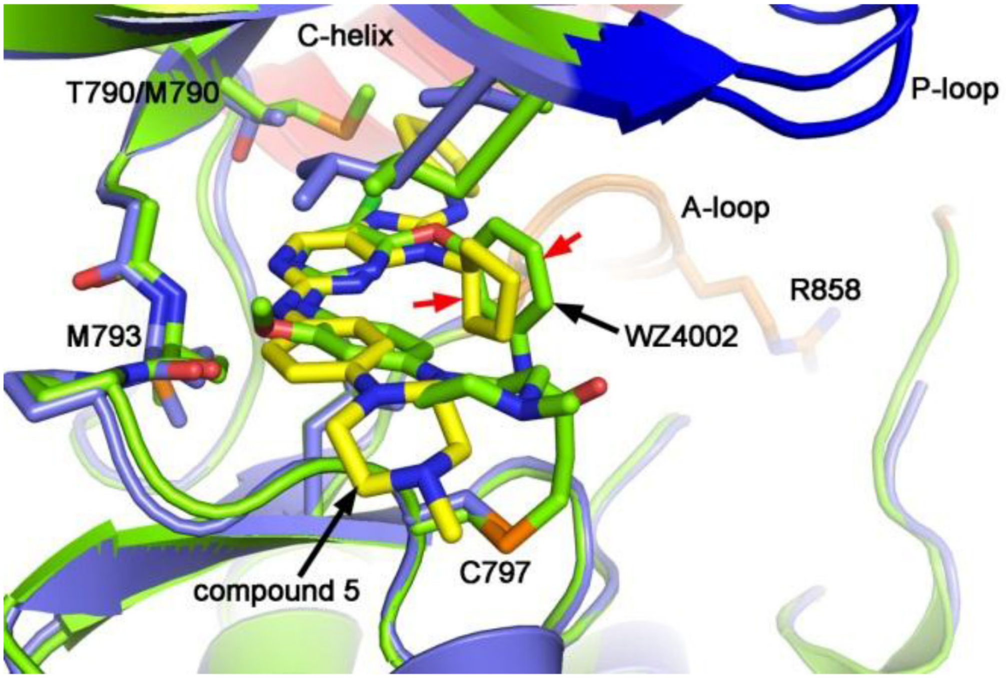

Supplementary Figure 4: Superimposition of EGFR T790M/WZ4002 with L858R/SKLB(5). EGFR L858R and T790M structures are shown as cartoons in slate and green, respectively. SKLB(5) and WZ4002 are shown as sticks in yellow and green, respectively. It can be seen that the 4-phenoxyl moiety in WZ4002 and 9-cyclopentyl moiety in SKLB(5) occupy roughly the same position and interact with the hydrophobic clamp (red arrows).

Supplementary Table 1: Data collection and refinement statistics of complex crystal structure of EGFR L858R with SKLB(3), (5) and (6)

	L858R + SKLB(3)	L858R + SKLB(5)	L858R + SKLB(6)
**PDB ID**	5X26	5X27	5X28
**Data collection**			
Space group	I23	I23	I23
Cell dimensions			
a, b, c (Å)	145.3, 145.3, 145.3	145.4, 145.4, 145.4	145.5, 145.5, 145.5
α, β, γ (°)	90.0, 90.0, 90.0	90.0, 90.0, 90.0	90.0, 90.0, 90.0
Resolution (Å)	50.0–2.95 (3.18–2.95)	50.0–2.95 (3.18–2.95)	50.0–2.95 (3.18–2.95)
R_{merge}	0.071 (0.413)	0.078 (0.416)	0.067 (0.426)
I/σ	14.6 (3.4)	16.6 (3.4)	14.8 (2.2)
Completeness (%)	98.2 (97.9)	96.4 (98.7)	99.1 (98.1)
Redundancy	3.1 (2.9)	5.3 (5.3)	3.2 (2.7)
**Refinement**			
Resolution (Å)	38.8–2.95	45.97–2.95	31.0–2.95
No. reflections	10697	10536	10848
R_{work}/R_{free}	0.196/0.222	0.203/0.239	0.209/0.250
No. atoms			
Protein	2372	2376	2410
Ligand/ion	34	36	37
Water	38	31	42
B-factors			
Protein	59.6	60.4	68.1
Ligand/ion	60.7	59.2	68.0
Water	54.7	57.3	62.8
R.m.s. deviations			
Bond lengths (Å)	0.014	0.015	0.013
Bond angles (°)	1.277	1.339	1.235
Ramachandran Plot			
Favored regions	288 (97.30%)	286 (96.62%)	295 (97.04%)
Allowed regions	8 (2.70%)	9 (3.04%)	9 (2.96%)
Outliers	0 (0.00%)	1 (0.34%)	0 (0.00%)

§Values in parentheses are for highest-resolution shell.

Supplementary Table 2: Data collection and refinement statistics of complex crystal structure of EGFR T790M/V948R with SKLB(3), (5) and (6)

	**T790M/V948R + SKLB(3)**	**T790M/V948R + SKLB(5)**	**T790M/V948R + SKLB(6)**
**PDB ID**	5X2A	5X2C	5X2F
**Data collection**			
Space group	P21	C2	P21
Cell dimensions			
a, b, c (Å)	72.2, 104.0, 87.2	155.8, 72.4, 76.2	71.5, 102.6, 87.0
α, β, γ (°)	90.0, 101.4, 90.0	90.0, 113.4, 90.0	90.0, 102.6, 90.0
Resolution (Å)	50.0–1.85 (1.92–1.85)	50.0–2.05 (2.12–2.05)	50.0–2.20 (2.28–2.20)
Rmerge	0.074 (0.593)	0.082 (0.482)	0.106 (0.566)
I/σ	14.6 (2.3)	11.8 (2.0)	8.9 (2.1)
Completeness (%)	98.0 (96.4)	98.7 (98.0)	89.4 (79.8)
Redundancy	3.5 (3.3)	2.7 (2.4)	3.2 (2.9)
Refinement			
Resolution (Å)	39.5–1.85	37.9–2.05	39.2–2.20
No. reflections	105211	48202	55505
R_{work} / R_{free}	0.174/0.219	0.175/0.202	0.202/0.240
No. atoms			
Protein	9893	4960	9707
Ligand/ion	208	111	144
Water	1003	526	380
B-factors			
Protein	29.6	33.8	36.6
Ligand/ion	34.7	39.9	32.8
Water	39.0	41.5	38.1
R.m.s. deviations			
Bond lengths (Å)	0.023	0.017	0.014
Bond angles (°)	1.890	1.594	1.325
Ramachandran Plot			
Favored regions	1150 (98.21%)	561 (97.57%)	1183 (98.26%)
Allowed regions	21 (1.79%)	12 (2.09%)	20 (1.66%)
Outliers	0 (0.00%)	2 (0.35%)	1 (0.08%)

[§]Values in parentheses are for highest-resolution shell.

Supplementary Table 3: Data collection and refinement statistics of complex crystal structure of EGFR T790M with WZ4003

	T790M + WZ4003
PDB ID	5X2K
Data collection	
Space group	I23
Cell dimensions	
a, b, c (Å)	149.3, 149.3, 149.3
α, β, γ (°)	90.0, 90.0, 90.0
Resolution (Å)	50.0–3.20 (3.45–3.20)
Rmerge	0.041 (0.415)
I/s	20.5 (2.0)
Completeness (%)	100.0 (100.0)
Redundancy	19.7 (19.4)
Refinement	
Resolution (Å)	43.1–3.20
No. reflections	8939
Rwork / Rfree	0.226/0.244
No. atoms	
Protein	2421
Ligand/ion	35
Water	9
B-factors	
Protein	61.3
Ligand/ion	61.2
Water	44.7
R.m.s. deviations	
Bond lengths (Å)	0.009
Bond angles (°)	1.082
Ramachandran Plot	
Favored regions	298 (97.39%)
Allowed regions	8 (2.61%)
Outliers	0 (0.00%)

§ Values in parentheses are for highest-resolution shell.
